# The effectiveness of interventions to change six health behaviours: a review of reviews

**DOI:** 10.1186/1471-2458-10-538

**Published:** 2010-09-08

**Authors:** Ruth G Jepson, Fiona M Harris, Stephen Platt, Carol Tannahill

**Affiliations:** 1 Department of Nursing and Midwifery, University of Stirling, Stirling, FK9 4LA, UK; 2 Nursing Midwifery & Allied Health ProfessionsResearch Unit, University of Stirling, Stirling, UK; 3 Centre for Population Health Sciences, University of Edinburgh, Edinburgh, EH8 9AG, UK; 4 Glasgow Centre for Population Health, 94 Elmbank Street, Glasgow, G2 4DL, UK

## Abstract

**Background:**

Several World Health Organisation reports over recent years have highlighted the high incidence of chronic diseases such as diabetes, coronary heart disease and cancer. Contributory factors include unhealthy diets, alcohol and tobacco use and sedentary lifestyles. This paper reports the findings of a review of reviews of behavioural change interventions to reduce unhealthy behaviours or promote healthy behaviours. We included six different health-related behaviours in the review: healthy eating, physical exercise, smoking, alcohol misuse, sexual risk taking (in young people) and illicit drug use. We excluded reviews which focussed on pharmacological treatments or those which required intensive treatments (e.g. for drug or alcohol dependency).

**Methods:**

The Cochrane Library, Database of Abstracts of Reviews of Effectiveness (DARE) and several Ovid databases were searched for systematic reviews of interventions for the six behaviours (updated search 2008). Two reviewers applied the inclusion criteria, extracted data and assessed the quality of the reviews. The results were discussed in a narrative synthesis.

**Results:**

We included 103 reviews published between 1995 and 2008. The focus of interventions varied, but those targeting specific individuals were generally designed to change an existing behaviour (e.g. cigarette smoking, alcohol misuse), whilst those aimed at the general population or groups such as school children were designed to promote positive behaviours (e.g. healthy eating). Almost 50% (n = 48) of the reviews focussed on smoking (either prevention or cessation). Interventions that were most effective across a range of health behaviours included physician advice or individual counselling, and workplace- and school-based activities. Mass media campaigns and legislative interventions also showed small to moderate effects in changing health behaviours.

Generally, the evidence related to short-term effects rather than sustained/longer-term impact and there was a relative lack of evidence on how best to address inequalities.

**Conclusions:**

Despite limitations of the review of reviews approach, it is encouraging that there are interventions that are effective in achieving behavioural change. Further emphasis in both primary studies and secondary analysis (e.g. systematic reviews) should be placed on assessing the differential effectiveness of interventions across different population subgroups to ensure that health inequalities are addressed.

## Background

Chronic diseases, such as cancers, cardiovascular diseases (CVD), diabetes, and respiratory diseases, account for 59% of the 57 million deaths annually and 46% of the global burden of disease [1]. In 2002, the World Health Report [2] identified a number of important lifestyle risk factors for such diseases, including physical inactivity; diet-related factors and obesity; and the use of addictive substances such as tobacco, alcohol and illicit drugs.

These lifestyle factors have significant effects on mortality and morbidity, particularly in industrialised countries. For example, data for the WHO European Region show that physical inactivity is a risk factor for diseases such as cardiovascular diseases, non-insulin- dependent diabetes, hypertension, some forms of cancer, musculoskeletal diseases and psychological disorders. These diseases are estimated to account for nearly 600,000 deaths per year [3]. Similarly, obesity and being overweight are risk factors for diseases such as type 2 diabetes, certain types of cancer and cardiovascular diseases, and affect between 30% and 80% of adults and up to one third of children [4]. Alcohol is also a significant cause of mortality: alcohol-related deaths increased by 15% between 2000 and 2002, and now represent 6.3% of all deaths in the European Region [5].

Sexual risk taking and drug misuse also significantly contribute to ill health and have negative effects on well being among young people. In 28 high income (OECD) nations at least 1.25 million teenagers become pregnant each year; of these, approximately 40% (half a million) will seek to terminate the pregnancy while the other 60% (three quarters of a million) will become teenage mothers [6]. The United States has the highest teenage birth rate in the developed world and the United Kingdom has the highest teenage birth rate in Europe [6]. Worldwide, young people (15-24 years) have the highest rate of sexually transmitted infections (STIs) of any age group. Up to 60% of the new infections and 50% of all people living with HIV globally are in this age group [7].

Most public health and health promotion interventions - whether they focus on the individual, community, whole populations or the environment - seek in some way to change health behaviour by changing health-related knowledge, attitudes and/or structural barriers and facilitators [8]. Social psychological theories such as social cognition theory are commonly used in the development of interventions [9]. Key elements of such theories include knowledge of health risks, perceived self efficacy, goals and motivations and barriers and facilitators [10]. Most health promotion interventions include one or more of the following components: education and knowledge building (around the health issue); motivation and goal setting (e.g. alcohol brief interventions and counselling); and community-based techniques to encourage a change in behaviour or reduce structural or cultural barriers. These interventions can be delivered at three different levels, which we explore below: individual, community and population level interventions. Individually targeted interventions are usually aimed at those with an existing 'risky' behaviour such as smoking or alcohol misuse. Community level interventions focus on particular population groups such as people in a particular workplace or young people in schools. Finally, population level interventions tend to rely on the use of mass media activities, policies or legislation.

All three levels of intervention are aimed at achieving changes in lifestyle, as well as improving knowledge and influencing attitudes towards positive healthy behaviours. However, there is a need to take into account the socio-economic and cultural contexts within which they are located. For instance, an intervention to promote healthy eating within an affluent locality might involve a rather different approach from one undertaken in an area of low income and high unemployment.

In light of the growing concern around the link between 'negative' health behaviours and ill health, we were commissioned by the Public Health section of the UK's National Institute of Health and Clinical Excellence (NICE) under the Behaviour Change Programme Development Group to review the relevant evidence in this area. This paper is an update of the findings of this 'review of reviews' of interventions to change health behaviours [11]. It was one type of evidence used to develop NICE public health programme guidance on behaviour change [12].

Our aim was systematically to collate, evaluate and synthesise review-level findings on the effectiveness of interventions to change unhealthy behaviours or promote healthy behaviours. This synthesis was intended to provide researchers, policy planners, decision-makers and practitioners with an accessible, good quality overview of the evidence in these topic areas. The review focused on six groups of behaviour change interventions:

• Interventions to encourage people to quit tobacco use

• Interventions to reduce heavy alcohol use

• Interventions to encourage physical activity

• Interventions to encourage healthy eating (excluding diets for weight loss)

• Interventions to prevent or reduce illicit drug use (excluding drug dependency)

• Interventions to prevent or reduce sexual risk taking in young people.

A subsidiary aim of the review was explore, where possible, the evidence of impact of interventions on health inequalities.

## Methods

### Inclusion criteria

#### 1) Types of reviews

a) Systematic reviews and meta-analyses published between 1995 and 2008 (reviews published before this time are likely to be out of date)

b) English language reviews as we were constrained by time and resource issues. (However, many of the included English language reviews contained primary studies in languages other than English.)

c) Cochrane reviews and systematic reviews in the Database of Abstracts of Reviews of Effects (DARE), which encompasses reviews gathered from searching a wide range of OVID databases

d) Other good quality reviews which have a low risk of bias (see section on quality assessment)

e) Less robust systematic reviews in areas where no other evidence exists.

#### 2) Content of the reviews

Two sets of inclusion and exclusion criteria were applied in the selection process: those that applied across all the health behaviours (see Table 1); and those that were specific to particular health behaviours (Table 2). For the six specific health behaviours of interest, interventions aimed at either preventing or delaying onset of the health behaviour were included, as well as those aimed at helping people to change an existing behaviour. However, interventions aimed at treating alcohol or drug dependency were not included as they were considered to require more intensive types of treatments, hence different forms of intervention. Healthy eating and physical activity were limited to outcomes related to changes in knowledge, attitudes or behaviour but did not include outcomes such as weight loss, weight reduction, nor programmes of obesity treatment or exercise specifically targeting high risk groups such as people with cardiovascular disease or cancer. Reviews of the following interventions were also excluded: health screening; psychiatric interventions as part of treatment for those with mental illness; interventions with only a clinical or pharmacological focus (e.g. reducing risk of heart disease); interventions carried out within secondary or tertiary care; drug interventions (including the use of vitamin supplements for healthy diets); and interventions aimed at treating alcohol or drug dependency.

**Table 1:** Inclusion and exclusion criteria which applied across the six health behaviours

*Inclusion Criteria*
1. Systematic reviews and meta-analyses

2. English language publications only

3. Focus on public health, health promotion (or related research) or primary care led interventions which contained an educational and/or behavioural component

4. Year of publication limited to 1995-2006

*Exclusion Criteria*

1. Reviews of health screening

2. Reviews of psychiatric interventions as part of treatment of those with mental illness

3. Reviews of interventions with only a clinical or pharmacological focus (e.g. reducing risk of heart disease, diet for diabetes care etc).

4. Reviews of interventions carried out within secondary or tertiary care

5. Reviews of drug interventions

6. Review of interventions which did not contain a behavioural/educational component to the intervention

7. Reviews of interventions which did not have the aim of changing any of the six health behaviours

**Table 2:** Inclusion/exclusion criteria for each of the six health behaviours

Health Behaviour	Population	Intervention	Exclusions	Outcomes
***Smoking***	Smokers Those with raised risk (e.g. pregnant women) General population	Interventions with a behavioural or educational component; advertising/media campaign; smoke free policies; other (to promote the outcomes)	Nicotine replacement therapy; other drug therapies; acupuncture; interventions aimed at treatment of smoking-related illness	Change in behaviours Smoking cessation; smoking prevention

***Physical activity***	Those with raised risk (e.g. overweight, sedentary or pregnant) General population	Interventions with a behavioural or educational component; advertising/media campaign; other (to promote the outcomes)	Interventions aimed at treating health problems (e.g. arthritis, back pain and intermittent claudication)	Change in behaviours Prevention of health problems related to sedentary lifestyle; increased uptake of exercise; increase in exercise levels

***Alcohol misuse***	Problem drinkers Those with raised risk (e.g. pregnant women) General population	Interventions with a behavioural or educational component; advertising/media campaign; interventions to reduce drink driving; other (to promote the outcomes)	Programmes to maintain abstinence; reviews of those with alcohol dependence	Change in behaviours Prevent/reduce alcohol consumption; prevent/reduce drink driving; promote moderate drinking

***Diet***	General population	Interventions with a behavioural or educational component; advertising/media campaign; Must have the aim of promoting healthy eating, rather than solely focussing on weight loss;	Interventions aiming only to reduce risk factors (blood pressure; hypertension); population with a chronic disease (e.g. those with diabetes or heart disease) Weight loss diets without a behavioural/educational component	Change in behaviours Dietary change

***Illicit drug use***	General population	Interventions with a behavioural or educational component; advertising/media campaign; other (to promote the outcomes)	Interventions aimed at illicit drug misusers (i.e. those with a dependency on illicit drugs)	Change in behaviours Prevention of illicit drug use

***Sexual risk taking in young people***	Young people	Interventions with a behavioural or educational component; advertising/media campaign; other (to promote the outcomes)	Interventions aimed at sexual risk takers (e.g. treatments for STIs; pregnancy counselling)	Change in behaviours Reduction of sexual risk taking; reduction of STDs; reduction of teenage pregnancy rates

### Search strategy

Searches were initially conducted in February 2006 for Cochrane and other systematic reviews and updated in 2008, as detailed below. As a starting point to identify the highest quality review level evidence, the Cochrane Database of Systematic Reviews (CDSR) was searched to identify Cochrane Reviews and the Database of Reviews of Effectiveness (DARE) was used for non-Cochrane reviews. DARE includes published and unpublished systematic reviews that have been assessed according to strict quality criteria by the Centre for Reviews and Dissemination (CRD) in York, UK. DARE represents an excellent resource since it includes quality assessed systematic reviews sourced by monthly searches of a wide range of electronic databases. As a final check to reveal what more recent reviews might be missed through this strategy, we also ran searches for each of the six public health topics on a range of OVID databases: AMED, ERIC, Cinahl, EmBase, Medline and PsycINFO. These searches were restricted by terms to identify reviews only, including 'meta-analysis', 'evidence-based review' or 'systematic review'. Full search histories are available on request from the corresponding author.

These searches generated a total of 2709 potentially relevant reviews. The search was updated in November 2008 by searching The Cochrane Library and DARE for new and updated Cochrane reviews and other high quality systematic reviews published since February 2006. This yielded a further 16 new reviews (out of 30 identified through the search), and 12 Cochrane reviews which had been updated since the initial search.

### Applying inclusion criteria

**Figure 1:**
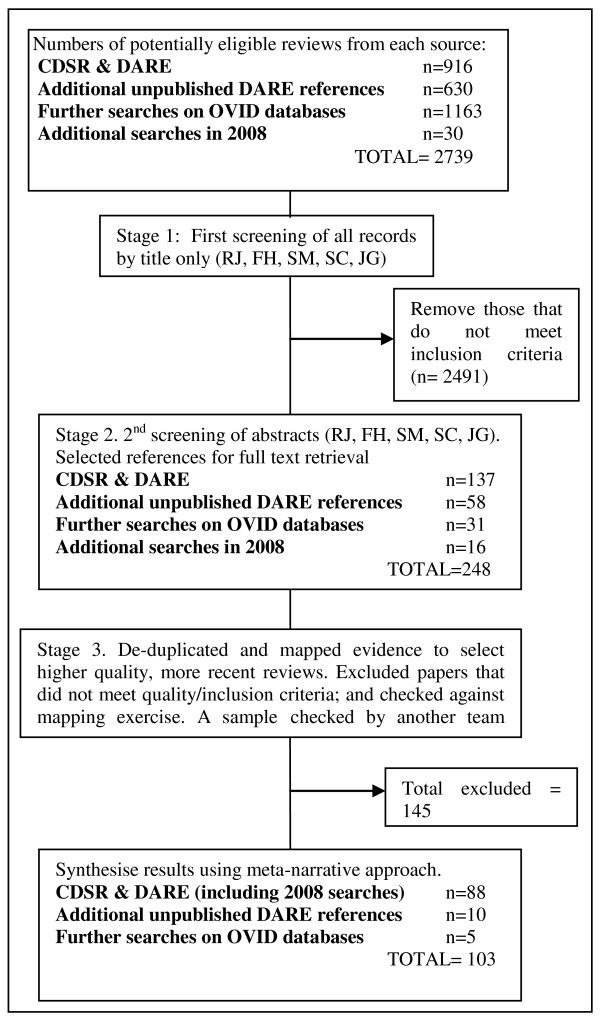
**Quorum statement**.

Titles and abstracts were independently screened by two reviewers. Any discrepancies in selections were discussed until consensus was reached. Another stage of screening consisted of a mapping exercise, where references were mapped into categories of evidence and two reviewers agreed to include or exclude further references based on the quality of the reviews and the date of publication. Since Cochrane reviews are usually the most comprehensive and of high quality, they were selected if there was more than one review in a particular topic area. Other reviews were selected on the basis of most recent publication date and quality of the review (see below for details of how we assessed quality of the reviews). The full review process is illustrated in the quorum statement (see Figure 1).

### Quality assessment

Potentially relevant reviews were assessed for quality using a checklist adapted from the NICE 'Methods for the development of NICE public health guidance' [13]. We prioritised reviews that had a transparent and replicable data search methodology and analysis. We scored reviews as "++" if at least 10 specified criteria were met, "+" if at least seven criteria were met, and "-" if fewer than seven criteria were met (Table 3). We also scored reviews on the type of evidence they were reviewing, such as RCTs or non-RCTs (see Table 4). The classification of bias (e.g. ++) was then combined with the type of evidence (e.g. 1) to give a level of evidence. For example, high-quality meta-analyses or systematic reviews of RCTs were coded as 1++. All data extracted on quality were extracted by one reviewer and checked by a second member of the team. Any discrepancies in the data that were extracted (e.g. differences in scoring) were resolved by discussion

**Table 3:** Criteria used to determine the potential for bias in the reviews

	Criteria which had to be met	
Criteria	++	+
1. Was there a focused aim or research question?	Yes	Yes

2. Explicit inclusion/exclusion criteria	Yes	Yes

3. More than 1 assessor/selector	Yes	

4. Provide details of databases searched	Yes	Yes

5. Lists years searched	Yes	Yes

6. Followed up references in bibliographies	Yes	

7. Experts consulted for further sources		

8. Grey literature included/searched		

9. Specified search terms/strategy	Yes	Yes

10. Not restricted to English language papers only	Yes	

11. Quality assessed	Yes	Yes

12. Data supports conclusions	Yes	Yes

**Table 4:** Scoring by type of evidence included in the reviews

Classification	Type of evidence
1	Systematic reviews of RCTs

2	Systematic reviews of individual, non-RCTs, case-control studies, cohort studies, controlled before-and-after (CBA), interrupted time series (ITS), correlation studies

1&2	Systematic reviews of **both **RCTs and non-RCTs, case-control studies, cohort studies, controlled before-and-after (CBA), interrupted time series (ITS), correlation studies

### Data extraction

Data were extracted by one of four people, and a sample checked by another member of the team. No formal synthesis (such as meta-analysis) was undertaken: a narrative summary of the results was more appropriate for a review of reviews.

## Results

We identified 103 systematic reviews evaluating interventions aimed at changing health behaviour in one or more of the six areas. Some of these reviews covered several behaviours. The reviews included studies which targeted specific individuals or organisations (e.g. through counselling within education) or more generally (e.g. mass media interventions or legislation).

We synthesised the results under three research areas:

1. Evidence for the effectiveness of interventions to prevent, reduce or promote the six health behaviours

2. Evidence for the effectiveness of interventions across several health behaviours

3. Evidence for the effectiveness of interventions in targeting health inequalities.

Full consideration of such a large number of reviews would prove too lengthy for this paper, therefore we have chosen to highlight the main findings rather than provide details of individual interventions discussed within each paper. A fuller version of the original document is available from the authors on request. Table 5 summarises the quality of the included reviews and Table 6 provides a brief overview of the studies, grouped by level of intervention (population, community or individual).

**Table 5:** Quality of the reviews by type of health behaviour

Health Behaviour	High quality reviews			Well conducted reviews			Reviews of variable quality			Total
Type of evidence* Potential for bias**	1 ++	1&2 ++	2 ++	1 +	1&2 +	2 +	1 -	1&2 -	2 -	

**Smoking**	8	3	0	14	10	1	6	3	3	**48**

**Physical Activity**	5	1	2	2	7	1	2	1	1	**24**

**Alcohol**	3	2	0	0	3	1	1	1	3	**15**

**Healthy eating**	0	0	0	1	12	0	0	1	0	**13**

**Illicit drug use**	1	1	0	0	0	0	0	1	1	**4**

**Sexual risk taking**	1	1	0	1	0	0	2	3	0	**8**

Notes: columns are not totalled because some reviews were included in more than one health behaviour

*Study designs included within the systematic reviews: 1 = RCT; 2 = non-RCT; 1&2 = include both study designs.

** ++ = low potential; - = high potential

**Table 6:** Summary of reviews for each of the health behaviours

Health Behaviour	Total number	No. aimed at general population	No. aimed at community, school or work places	No. aimed at targeted populations or individuals
**Smoking**	**48**	5	6	Young people (11) Smokers (26)

**Physical Activity**	**24**	4	1	Young people (6) Older people (2) Adults over 18 years(8)

**Alcohol**	**15**	4	0	Young people (2) Pregnant women (1) Drink drivers (3) Problem drinkers (4)

**Healthy eating**	**13**	0	3	Young people (4) Older people (1) Pregnant women (1) Non specific (6)

**Illicit drug use in young people**	**4**	0	4	0

**Sexual risk taking in young people**	**8**	0	8	0

Notes: columns are not totalled because some reviews were included in more than one health behaviour

Some reviews included more than one population group, so the rows may not add up to the total number of reviews for each health behaviour

### 1. Evidence for the effectiveness of interventions to prevent, reduce or promote each of the six health behaviours

The focus of interventions varied, depending on the target population. Interventions targeting individuals generally aimed to change an existing behaviour such as cigarette smoking or alcohol misuse, whilst interventions targeting workplaces, schools or the general population were often more focused on promoting positive behaviours (e.g. healthy eating or exercise).

#### 1.1. Smoking and tobacco use

We identified 48 systematic reviews which evaluated interventions to aid smoking cessation, prevent relapse or prevent people taking up smoking [14-61]. One further review evaluated population-level tobacco control interventions and their effect on social inequalities [62]. This review is discussed in more detail in section 3.

Eleven reviews evaluated interventions aimed at the prevention of smoking, promoting smoking cessation or reducing smoking prevalence in young people [22,27,29,30,40,46,50,51,53,56,60,61]. There is some evidence that mass media interventions can be effective in preventing the uptake of smoking in young people, but overall the evidence is not strong. Information provision interventions alone are not effective and there is only limited evidence for the effects of interventions that mainly seek to develop social competence.

There is little evidence of effectiveness of other interventions, such as reducing tobacco sales to minors. Interventions with retailers can lead to large decreases in the number of outlets selling tobacco to youths but there is insufficient evidence to say whether this is linked to reduction or cessation of smoking in young people.

Twenty six of the 48 systematic reviews evaluated interventions aimed at achieving positive changes in tobacco use in adults known to use tobacco (i.e. targeting smokers). Of these, twenty-two of these evaluated interventions for adult cigarette smokers in general, two focussed specifically on pregnant and postpartum women [25,36], and two evaluated interventions for *smokeless *tobacco use [24,38]. The following sections describe the range of interventions to reduce tobacco consumption in individuals and are grouped by their effectiveness.

Interventions which show a positive effect include advice from health professionals, the rapid smoking form of aversion therapy, self help materials, telephone counselling (compared to less intensive interventions), nurse-delivered interventions, group counselling (which is also more effective than self help), and oral examination and feedback for reducing smokeless tobacco use. However, there is no evidence for the effectiveness of interventions targeting waterpipe smokers. Interventions to promote smoking cessation or smoking reduction with pregnant women are generally effective across the range of intervention types, indicating that pregnancy may be a point in the lifecourse when positive behaviour change can be achieved.

There is less clear or inconclusive evidence of effectiveness for social support interventions (e.g. buddy systems or friends and family support), relapse prevention, biomarker feedback or biomedical risk assessment, exercise, Internet and computer-based interventions and interventions by community pharmacy personnel or dentists. Currently there is not enough evidence to show which interventions are most effective for decreasing parental smoking and preventing exposure to tobacco smoke in childhood.

Interventions for which there is no evidence of effectiveness include hypnotherapy and interventions based on the transtheoretical model of change. The latter proposes that interventions designed to take into account an individual's current stage of change (or readinesss to change a health behaviour) will be more effective and efficient than "one size fits all" interventions [63]. The model assumes that people move through six changes of change, from 'pre-contemplation' through to 'termination' (when the behaviour has successfully been changed). However, this assumption does not sit comfortably within wider theories of social change, which posit that change rarely moves in a linear fashion. Additionally, a systematic review exploring a range of 'stage based' interventions for smoking cessation found little evidence of effectiveness [45].

Six studies evaluated smoking interventions that were undertaken in either workplace or community settings [19,26,33,49,64,65]. Interventions which show an effect in the workplace include those aimed at encouraging individuals to quit. The results are consistent with those found in other settings [64]. Particularly effective interventions include individual and group counselling and pharmacological treatment to overcome nicotine addiction. Self-help materials are less effective, and competitions and incentives, while increasing attempts to stop smoking, were not consistently found to increase the rate of quitting. Interventions aimed at the wider community included multi-component interventions and those which use multiple channels to provide reinforcement, support and norms for non-smoking. These show limited effectiveness.

Five systematic reviews evaluated interventions aimed at the general population to prevent the uptake of smoking or reduce smoking rates [15,16,28,47,66]. Mass media interventions show evidence of a small effect in preventing the uptake of smoking, but the evidence comes from a heterogeneous group of studies of variable methodological quality. Smoking cessation interventions that show some evidence of effectiveness include 'Quit and Win' contests and policies to reduce smoking in public places. However, policy interventions are normally evaluated using non-controlled designs (e.g. before and after studies), which makes it difficult to determine the extent to which the outcomes could be attributed to the intervention.

#### 1.2. Physical activity

Twenty-four systematic reviews evaluated interventions to increase or promote the uptake of physical activity [23,39,58,67-85]. Six of these explored the effectiveness of interventions to increase physical activity in young people [70,78,83,85-87]. There is moderate evidence of effectiveness for curriculum-based activities in schools. The most effective school-based physical activity interventions include printed educational materials and curricula that promoted increased physical activity during the whole day (i.e., recess, lunch, class-time, and physical education classes). The most effective non-curricular school activities include education and provision of equipment for monitoring TV or video-game use; engaging parents in supporting and encouraging their children's physical activity; and those implemented during school breaks (painting school playgrounds, playground supervisors implementing a games curriculum, and taught playground games or introduced equipment). There is no evidence of an effect of other non-curricular activities, such as active travel to school, extra-curricular activities and summer schools or camps.

The most recent review reported strong evidence that school-based interventions with involvement of the family or community and multi-component interventions can increase physical activity in adolescents [85].

Ten systematic reviews evaluated targeted interventions aimed at increasing physical activity for adults. Eight of these evaluated interventions for adults over 18 years [23,39,71,72,74,75,88,89], while two evaluated interventions specifically for the older population [69,84]. The interventions included the use of pedometers, telephone counselling, and professional advice and guidance (with continued support). Most of the reviews found some evidence of moderate effectiveness in the short term (less than three months) in increasing physical activity, but effects are not necessarily sustained over a longer time period (e.g. twelve months). Many of the studies were limited by the recruitment of motivated volunteers, and no studies examined the effect of interventions on participants from varying socioeconomic or ethnic groups. In addition, even those interventions which are moderately effective in increasing exercise did not necessarily meet a predetermined threshold of physical activity. These findings were also supported by the findings from reviews of interventions for the older population, which found a small but short-lived effect of home-based, group-based and educational physical activity interventions on increasing physical activity.

Physical activity interventions for which there is inconclusive evidence include biomarker feedback and brief motivational interventions. In addition, there is no evidence that interventions based on the stages of change model increase levels of physical activity.

One systematic review evaluated physical activity programmes in the workplace [82], finding evidence of a moderate effect on increasing physical activity levels. Interventions comprised self-help or educational programmes, and exercise programmes involving aerobics, walking, jogging, swimming, cycling, muscle strengthening, endurance, flexibility and stretching.

Four systematic reviews evaluated interventions aimed at increasing physical activity in the general population. Two evaluated interventions to increase participation in sport [76,90], one evaluated interventions to promote walking and cycling [81], and one evaluated mass media interventions [73]. However, the first two reviews found that no studies had been undertaken to identify any intervention designed to increase active and/or non-active participation in sport (including policy interventions). There is evidence that targeted behaviour change programmes can be effective in changing the transport choices of motivated subgroups, but the social distribution of their effects and their effects on the health of local populations are unclear. Evidence of effectiveness of other types of intervention is inconsistent, of low validity, based on single, highly contextualised studies, or non-existent. There is evidence (with a higher risk of bias) that mass media interventions may increase physical activity, but the effects tend to be in small subgroups or for specific behaviours, such as walking.

#### 1.3. Alcohol misuse

Fifteen reviews evaluated a range of interventions aimed at reducing alcohol consumption in problem drinkers, preventing or delaying the onset of alcohol use in young people, or reducing dangerous activities associated with drinking (e.g. drink-driving) [91-105]. No consistent definitions of what constitutes harmful alcohol consumption were available from existing guidelines or research; however, it is commonly held that behavioural interventions are appropriate for mild to moderate alcohol consumption or binge drinking, whereas more severe problems, such as alcohol dependency, may require specialist addiction treatment. Interventions for the latter were excluded from the review.

Two reviews evaluated interventions targeting school children [95,96]. There is evidence of a positive effect of school-based instructional programmes for reducing riding with drivers under the influence of alcohol. However, there is insufficient evidence to determine the effectiveness of these programmes for reducing drinking and driving. There is also insufficient evidence to determine the effectiveness of peer organisations (e.g. groups of students and/or staff who encourage others to refrain from drinking alcohol) and social norming campaigns (typically, public information programmes based on the assumption that children may overestimate the amount and frequency of their peers' alcohol consumption) to reduce alcohol use, due to the small number of available studies.

Several reviews evaluated interventions for adult problem drinkers. One review assessed home visits for pregnant women who were problem drinkers [93] and found insufficient evidence to recommend their routine use. Three reviews were of interventions aimed at reducing driving under the influence of alcohol [97,100,103]. For convicted drink drivers, there is evidence of an effect of alcohol interlock programmes (where the car ignition is locked until the driver provides an appropriate breath specimen), but the effect of other interventions is inconclusive due to the variable quality of the evidence. According to a Cochrane review [97] which evaluated the impact of increased police patrols for alcohol impaired drinking, most studies found that such patrols reduce traffic crashes and fatalities. However these conclusions were based on poor quality evidence.

Four further reviews evaluated interventions for problem drinkers in general [91,98,102,105]. There is evidence of a small positive effect of brief behavioural counselling interventions in reducing alcohol intake. The most recent Cochrane review [98] of brief interventions delivered to people attending primary care (1-4 sessions) found that, overall, such interventions lower alcohol consumption. When data were available by gender, the effect was clear in men at one year of follow up, but not in women. The authors concluded that longer duration of counselling probably has little additional effect.

Four systematic reviews evaluated mass media interventions [92,94] and legislative interventions [99,104] aimed at people who drink and drive. None of the reviews included evidence from RCTs, mainly because of the difficulty of conducting controlled trials in these areas. One well conducted review found insufficient evidence of effectiveness for mass media 'designated driver programmes' in increasing the number of designated drivers [92]. The other reviews reported that effective interventions for reducing alcohol and driving related outcomes included mass media campaigns [94]; low blood alcohol concentration laws for young drivers [104]; and a policy of a minimum legal drinking age (MLDA) of 21 years (which reduced traffic crashes and alcohol consumption) [99].

We did not identify any reviews which evaluated evidence relating to mass media interventions to promote 'safe' drinking levels or reduce 'risk drinking' (e.g. binge drinking).

#### 1.4. Healthy eating

Thirteen systematic reviews evaluating behavioural or psychological interventions to promote healthy eating were identified [23,58,79,106-115]. Overall there is evidence that interventions can change eating habits, at least in the short term.

Four reviews evaluated interventions targeted at children or young people [79,112-114]. There is evidence of an effect of interventions aimed at increasing fruit and vegetable intake in children aged 4-10 years and interventions for youth aged 11-16 years. However, there is insufficient evidence of an effect of interventions in pre-school children.

Three reviews evaluated community based interventions. One review reported evidence of a small effect of community interventions for people aged 4 years and above on increasing fruit and vegetable intake [107]. There is also evidence that interventions based in supermarkets are effective for promoting positive changes in shopping habits, although effectiveness was found to be confined only to the period during which the intervention took place [110]. Lastly one review evaluated community-level interventions for older people [109] but found little or no effect of interventions to increase fruit and vegetable intake.

Six reviews evaluated a range of targeted interventions or interventions aimed at individuals. There is evidence of a positive effect of stage-based lifestyle interventions delivered to a primary care population [58], telephone based interventions [108] and nutritional counselling interventions [106]. A review of interventions using a Mediterranean diet showed positive results for a range of outcomes, but it is not clear how the interventions brought about behaviour change [111].

There is inconclusive evidence as to the effectiveness of motivational interviewing [23] for changing eating behaviours. There is also inconclusive evidence for interventions such as health education, counselling, changes in environment and changes in policy, to encourage pregnant women to eat healthily [115].

#### 1.5. Illicit drug use

Only four reviews met our inclusion criteria for this section. There is more review level evidence relating to interventions aimed at treating drug users, which was specifically excluded under our search criteria.

All four reviews evaluated community-level interventions to prevent illicit drug use with young people [101,116-118]. The evidence base for this topic is limited and there are substantial gaps in knowledge. A positive effect of skill-based programmes in schools is demonstrated, but it is not possible to reach any conclusion about the effectiveness of non-school based programmes. There is also some evidence that the 11-13 age range may be a crucial period for intervention with vulnerable young people.

#### 1.6. Sexual risk taking in young people

Eight systematic reviews evaluating community based interventions were identified in this area. Four reviews focused on the reduction or prevention of HIV or other sexually transmitted infections (STIs) [119-122], while four evaluated sexual health promotion and the reduction or prevention of teenage pregnancies [123-126]. The reviews were of variable quality and most commented on the poor quality of existing primary studies, which made the process of synthesising evidence difficult. However, two conclusions can be drawn. First, in the area of risk reduction and prevention programmes, interventions are most effective in promoting the uptake of condom use, with some success in reducing the number of sexual partners and the frequency of sex. Second, interventions seeking to promote the use of contraception are more effective than interventions that promote abstinence. There was a single study of counselling to prevent or reduce teenage pregnancies, but the authors found that the available evidence was of such poor quality that they were unable to reach any firm conclusions about effectiveness.

### 2. Evidence to suggest that some interventions are effective/ineffective across the range of health behaviours

Many of the interventions included in this review were behaviour specific - e.g. aversion therapy for smoking cessation, tobacco bans and drink driver-related interventions. However, there were a few interventions - such as counselling and physician advice, mass media and motivational interventions - that were used across a range of behaviours. Table 7 outlines the interventions and their effectiveness across different behaviours.

**Table 7:** Research questions for future systematic reviews related to health inequalities

1. How does the effectiveness of interventions to effect positive changes in health behaviours vary according to the socio-economic, cultural or other characteristics of participants? This might include studies of effectiveness according to age, gender, ethnicity, social class and locality.
2. What is the effectiveness of interventions which address the interconnectedness of negative health behaviours? For instance, this review might explore the effectiveness of combined smoking and alcohol reduction/cessation interventions.
3. What is the demographic profile of those who gain access to interventions and how does this relate to existing knowledge on health inequalities? This review could explore the socio-economic characteristics of intervention participants to reveal the 'fit' between those groups most at risk from the ill effects of negative health behaviours and intervention participants.
4. What are the challenges for recruitment of intervention participants? What are the most effective ways of recruiting 'hard to reach' groups, such as ethnic minorities and the socially and economically disadvantaged?
5. What is the relationship between the outcomes of interventions and the social, cultural and demographic characteristics of participants? Questions to explore might include whether the intervention is equally effective with participants who differ with regard to gender, age, ethnicity, rural or urban location, and employment status.

### 3. Evidence for the effectiveness of interventions in targeting health inequalities

Despite the widely acknowledged link between social and economic inequalities and health, our review of reviews found no evidence which helped to develop an understanding of the following:

• Inequalities in levels of physical activity; alcohol misuse; healthy eating; illicit drug use; and sexual risk taking among young people.

• Inequalities in access to interventions to promote change in behaviour

• Inequalities in recruitment to interventions of 'hard to reach' groups

• Differential effectiveness of health behaviour interventions, which may result in increased health inequalities.

One review evaluated potential role of moderators (e.g. race, gender age, and setting) in the effectiveness of interventions to change the environment which had the aim of improving diet and/or activity in persons aged 3 to 18 years [87]. The authors found that only 17% of the 41 included studies looked at the effect of modifiers (gender being the most commonly studied and tested). The authors concluded that, *'Rather than being an exception, it is argued that tests of effect modifiers should become common practice in behavioral nutrition and physical activity research to increase our understanding of mechanisms of behavior change and to optimize interventions.'*

A further review explicitly explored the impact of population-level smoking cessation interventions on social inequalities [62]. It found that those in lower socio-economic groups, in manual occupations and those under the age of 25 years (particularly boys and 'non-white' young people) are more likely to be affected by increases in the price of tobacco products. However, it reported that there is also some evidence that price increases may be effective in people with a higher education. It also found that smoking restrictions in schools and restrictions on sales to minors may be more effective in girls than boys.

Most of the systematic reviews did not consider socio-economic differences or conduct sub-group analyses. Although some reviews may report socio-economic data, this does not reveal anything about inequalities unless there is an explicit attempt to explore data appropriately, for example whether there is differential take-up and/or effectiveness of the interventions according to variables such as gender, age and income.

## Discussion

A limitation of a review of reviews approach for knowledge synthesis might also be regarded as one of its strengths. Given the heterogeneity of interventions included within each review and the large number of papers across the six health behaviour areas, it has not been possible to report any particular area in great depth. While some readers might be frustrated by the lack of detail, one of the strengths of this review is its capacity to offer a broad overview of the evidence. We are able to bring to the attention of other researchers, policy makers and commissioners of research the areas where no systematic reviews have been conducted, and suggest potential research questions worthy of investigation at the review level. It was outside the scope of a review of reviews to determine whether this corresponded to gaps in primary data, or whether this reflected the research priorities/questions of systematic review authors.

Given the importance of socio-economic determinants of health and illness, and their impact on morbidity (as well as mortality), it is crucial that interventions designed to improve health take account of those very factors that may work against positive outcomes. As indicated above, this is one of the most significant gaps in the evidence base revealed by our study.

Until the mid 20^th ^century, infectious diseases were one of the main causes of mortality and morbidity in industrialised nations. Once these were largely brought under control, chronic diseases such as cancer and coronary heart disease assumed prominence. The 1970 s saw the emergence of a 'new' public health, highlighting the social and cultural factors which affect chronic diseases (e.g. lifestyle), and of new disciplines such as health promotion. Today the focus has shifted in line with the epidemiological transition, so there is increasing emphasis on social determinants of health rather than on infectious agents.

A series of reports on health inequalities been published in the UK (for instance, The Black Report [127], the Acheson Report [128] and the Wanless Report [129]). While the Black Report of 1980 focused primarily on material deprivation as a major explanatory factor in health inequalities, more recent research has explored the interplay between social position and the lifecourse in relation to the determinants of health [129-133,133]. The more recent change of focus from material deprivation towards a more multi-faceted understanding of determinants of health is important for interventions aimed at effecting changes in behaviour that may have an adverse affect on health. However, what is not clear is whether current health promotion initiatives have absorbed and embedded the 'determinants' approach in order to improve the health of those who might benefit most. It has been argued that the inverse care law is not always considered in relation to local provision of health promoting activities [134]. Indeed, it has been suggested that health promotion interventions frequently increase, rather than decrease, socioeconomic inequalities in health [128], since health promotion messages and interventions have a differential take-up across different social class groups. Those who are more affluent and have a higher level of formal education are more likely to modify their diets, give up smoking and increase levels of physical activity than are the less affluent with lower levels of formal education [135]. For instance, a mass media campaign to increase walking in Scotland ('Fitline') found that it had less appeal for those in the lower socioeconomic groups, despite higher awareness levels among these groups [136]. Furthermore, one-third of those who telephoned 'Fitline' to obtain further information were already regular exercisers, and may not have incurred any additional benefit.

In Tables 8 and 9 we have highlighted areas worthy of further synthesis. Despite the importance of socio-economic status as a determinant of health, few reviews explored socio-economic status or whether interventions had targeted those with the poorest health (e.g. those living in areas of disadvantage). Employment status, occupation, income level, gender, age, education, mobility and ethnicity are only some of the complex factors related to the determinants of health, and these do not include the more recent psychosocial [132] and lifecourse approaches [133]. Indeed, as Wilkinson argues, "The social consequences of people's differing circumstances in terms of stress, self-esteem and social relations may now be one of the most important influences on health" [137], pg 128]. We now understand how individuals become resilient to adverse circumstances (e.g. living in conditions of social deprivation) through, for instance, access to social support and strong social networks.

**Table 8:** Research questions for future systematic reviews on the effectiveness of interventions for health behaviour change

1. What is the effectiveness of interventions which target tobacco use or smoking cessation/reduction in older people?
2. What is the effectiveness of interventions which reduce or prevent alcohol misuse among older people?
3. What is the effectiveness of interventions to promote healthy eating at the population level? These might include mass media (e.g. TV, newspaper, billboard advertising, leaflet and poster advertising/information) or policy related interventions. Policy might include changes to food labelling to promote healthy eating.
4. What is the effectiveness of interventions targeting pregnant women to prevent illicit drug use?
5. What is the effectiveness of population level interventions, such as policy or legislation change or the use of mass media, to prevent illicit drug misuse? Further reviews are required more generally in the area of illicit drug use among adults and older people.

**Table 9:** Research questions for future systematic reviews on the effectiveness of interventions for health behaviour change

6. What is the effectiveness of interventions which target tobacco use or smoking cessation/reduction in older people?
7. What is the effectiveness of interventions which reduce or prevent alcohol misuse among older people?
8. What is the effectiveness of interventions to promote healthy eating at the population level? These might include mass media (e.g. TV, newspaper, billboard advertising, leaflet and poster advertising/information) or policy related interventions. Policy might include changes to food labelling to promote healthy eating.
9. What is the effectiveness of interventions targeting pregnant women to prevent illicit drug use?
10. What is the effectiveness of population level interventions, such as policy or legislation change or the use of mass media, to prevent illicit drug misuse? Further reviews are required more generally in the area of illicit drug use among adults and older people.

Studies that have explored the accumulation of risk of ill health over the lifecourse have highlighted the importance of social location and social capital. For instance, Schoon and Bynner [138], in a paper related to risk and resilience in young people, argue that interventions to improve health should aim to bolster incidences of positive adaptations to adverse circumstances rather than simply focusing on trying to put right negative behaviours. Linking into the health promotion agenda, they argue for the importance of primary prevention interventions, which take account of the context of the lives that an intervention seeks to improve, rather than simply taking a 'one size fits all' approach. However, there is little evidence at systematic review level that interventions to effect changes in behaviour are tackling the more complex interplay between health, illness and the wider determinants of health. As we noted above, this does not necessarily mean that the researchers in the primary studies themselves were unaware of a determinants approach. Nevertheless it is a significant finding that the systematic reviews covered here largely failed to take account of the inequalities agenda.

Research evidence clearly indicates that illness tends to cluster within lower socio-economic groups [139,140]. This means that it is even more crucial that those conducting systematic reviews (as well as those designing interventions) make health inequalities a central concern. As a recent WHO report states: "Together, the structural determinants and conditions of daily life constitute the social determinants of health and are responsible for a major part of health inequities between and within countries" [130].

The Cochrane Collaboration has a Cochrane Health Equity Field which aims to encourage reviewers to *'include explicit descriptions of the effect of the interventions not only on the whole population but to describe their effect upon the disadvantaged and/or their ability to reduce socioeconomic inequalities in health and to promote their use to the wider community.' *To aid reviewers in this task, they have developed an 'Equity Tool' which is available from their website http://equity.cochrane.org/en/index.html. We would urge reviewers to access this tool and include data on inequities in future publications.

Another notable finding is that there were many more reviews evaluating individual-level interventions than those at community or population level, which again may reflect practical/methodological concerns. What we were not able to ascertain was whether the lack of such reviews reflected a lack of primary population based intervention studies. However, as the success of mass media advertising campaigns in marketing consumer goods clearly demonstrates, population-level interventions certainly hold promise, despite not lending themselves to evaluation by traditional means, such as the randomised controlled trial.

Whatever the level or target of the intervention, one of the most significant challenges is to bring about a change in behaviour that is sustained over time. In the smoking cessation field this is dealt with through relapse prevention schemes. However, sustained change is a difficulty also faced by healthy eating and physical activity programmes, where the intervention may be highly effective for the duration of contact with participants but then become less effective and even lead to a reversal of positive change in behaviour over time.

Interventions aiming to achieve long-term, sustained behaviour change will require a different approach to evaluation. Currently, the majority of studies have a relatively short period of follow-up, with the longest usually no more than two years. In order to determine long-term effectiveness, there is a need to develop longitudinal studies that can run alongside the intervention and revisit the participants at several time points, charting the challenges to sustaining the healthy behaviours and learning from those who have successfully maintained new, healthful habits. This would enable policy makers and those delivering the interventions to gain a deeper understanding of the strengths and weaknesses of the intervention, with a view to improving the effectiveness of future interventions. Participatory and ethnographic approaches may be particularly suited to this form of evaluation.

### Limitations of the review methodology

Reviews are known as 'secondary' data sources because they collate and interpret original primary studies, and provide an interpretive overview of the collated findings. Reviews of reviews (such as the one reported here) bring together all of the systematic reviews in an area in order to provide an overview of the evidence. They involve a large body of high quality research literature, brought together in a systematic way, which can then be explored in more detail, if necessary, to answer particular research or clinical questions. Although 'reviews of reviews' are a useful endeavour for bringing together a large body of evidence and for investigating broad questions, several limitations should be acknowledged.

First, the evidence provided in such a review is 'twice removed' from the original primary data and the original primary research aims. Therefore, it is limited in its ability to provide detailed evidence of effectiveness for a particular intervention in a particular population group. Second, some of the high quality reviews might contain poor quality evidence (or very limited evidence), because that is all that is available. Third, even where no good quality reviews have been carried out in a particular area (e.g. mass media interventions for preventing illicit drug use), the absence of good primary evidence on that topic should not be assumed. There are many high quality primary studies that have not yet been synthesised into reviews. These primary studies cannot be included in a review of reviews, and often gaps in systematic review areas are thought to imply gaps in the primary evidence when this is not the case. Fourth, separate reviews may actually consider similar topic areas and therefore include a number of the same studies. Often the reviewers may have slightly different research questions, inclusion criteria and methods of analysis. Consequently, the results and conclusions may differ even though the included studies are very similar. Such differences are difficult to uncover and report within a review of reviews. Finally, when looking at the evidence in reviews, we were often limited by the questions that the authors of the reviews had decided are important and the outcomes that they included. These were often not the same questions that we have prioritised, even though the data might have been available in the primary research papers.

This review of reviews was also unable to identify the effectiveness of interventions which used comprehensive approaches (e.g. multi-component, working with a range of health behaviours). It is likely, however, that the effectiveness of the specific interventions reviewed would be enhanced through complementary supportive action. For example, behaviours are often interlinked (e.g. smoking and alcohol use; physical activity and healthy eating) and changing one behaviour may impact on one or more related behaviour.

## Conclusions

Despite the limitations of a review of reviews, we have gathered together a wide body of evidence which illustrates that there are many interventions that have effectively achieved behaviour change across a range of health behaviours. The interventions that appear to be most successful include workplace interventions to support smoking cessation, physical activity and healthy eating; school based interventions across the health behaviours; individual-level interventions drawing on physician advice to promote healthy eating, smoking cessation and responsible/safe levels of alcohol use; and counselling for tobacco and alcohol use. Pregnancy may be a point in the lifecourse when women are especially amenable to making health improving changes (particularly in smoking cessation, and physical activity).

Mass media interventions are relatively effective in addressing the interconnectedness of knowledge, attitudes and behaviour within health promoting social marketing. Similarly, population-level legislative interventions (such as smoking bans and age limits for alcohol use) tend to be a part of much wider, more comprehensive campaigns to secure public support for the underlying health promoting message. Clearly, interventions at the individual and community levels should recognise the importance of changing knowledge and attitudes along with promoting healthy behaviour. Although many of the interventions currently include educational components, few explicitly attempt to evaluate their success.

Systematic reviews should explore data from the primary studies showing how effectiveness varies in relation to social and economic difference (including wider determinants of health, such as gender, ethnicity and geographic location). Further research is needed to synthesise primary studies of the effectiveness of interventions across all six health behaviours to target those of lower socio-economic status who may be at highest risk of ill-health and who may engage in a range of unhealthy behaviours. For this reason, it is likely that health promoting interventions might benefit from a more multi-faceted approach: the effectiveness of the specific interventions reviewed here is likely to be enhanced through complementary supportive action. The inter-relationship between activities, such as alcohol use and smoking, may lend itself to a two-pronged intervention.

While there is a good body of high quality evidence of effectiveness of interventions to promote healthy behaviours, longer term follow-up is required in order to determine whether positive change is sustained and for how long.

## Competing interests

The authors declare that they have no competing interests.

## Authors' contributions

RJ was the Principal Investigator on the project, conceived the design of the study, involved in data extraction, analysis and drafting the manuscript. FH was involved in data extraction and analysis and drafting the manuscript. SP and CT were involved in the study as advisors and were also involved in drafting and commenting on the manuscript. All authors read and approved the final manuscript.

## Pre-publication history

The pre-publication history for this paper can be accessed here:

http://www.biomedcentral.com/1471-2458/10/538/prepub
